# Straintronic effect for superconductivity enhancement in Li-intercalated bilayer MoS_2_†

**DOI:** 10.1039/d0na00420k

**Published:** 2020-07-06

**Authors:** Poobodin Mano, Emi Minamitani, Satoshi Watanabe

**Affiliations:** Department of Materials Engineering, The University of Tokyo 7-3-1 Hongo Bunkyo Tokyo 113-8656 Japan watanabe@cello.t.u-tokyo.ac.jp; Institute for Molecular Science 38 Nishigo-Naka, Myodaiji Okazaki Aichi 444-8585 Japan eminamitani@ims.ac.jp

## Abstract

In this study, *ab initio* calculations were performed to show that the superconductivity in Li-intercalated bilayer MoS_2_ could be enhanced by applying either compressive or tensile strain. Moreover, the mechanism for superconductivity enhancement for the tensile strain case was found to be different than that of the compressive strain case. Enhanced electron phonon coupling (EPC) under tensile strain could be explained by an increase in the nesting function involved with the change in the Fermi surface topology in a wide range of Brillouin zones. The superconducting transition temperature *T*_c_ of 0.46 K at zero strain increased up to 9.12 K under a 6.0% tensile strain. Meanwhile, the enhancement in compressive strain was attributed to the increase in intrinsic electron phonon matrix elements. Furthermore, the contribution from interband scattering was large, which suggested the importance of electron pockets on the Fermi surface. Finally, 80% of the total EPC (*λ* = 0.98) originated from these pockets and the estimated *T*_c_ was 13.50 K.

## Abstract 2

We demonstrated that the superconductivity in Li-intercalated bilayer MoS_2_ can be enhanced by both compressive and tensile strains with different mechanisms.

## Graphical abstract

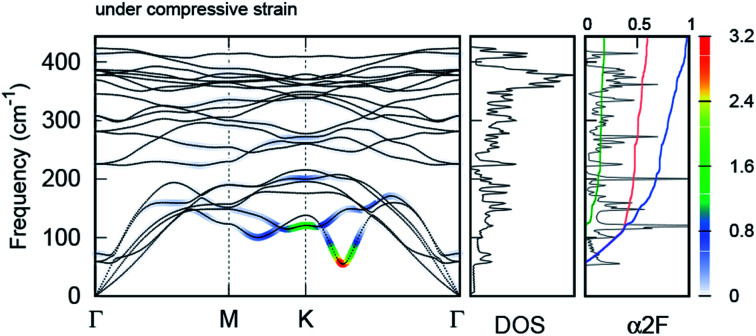

Layered materials have been investigated for a long time owing to their tunable features, which enable various applications. Amongst them, superconductivity has recently attracted considerable attention, and various mechanisms have been proposed to explain the emergence or enhancement of superconductivity in these layered materials.^1^ Graphene is a representative example of such materials. However, the small band gap in graphene limits its practical applications. Hence, MoS_2_, which is a transition metal dichalcogenide (TMD) with a band gap, has recently attracted significant attention.^2–6^ MoS_2_ becomes a metal by the intercalation of alkali metals.^7–9^ Furthermore, the intercalated bilayer MoS_2_ has been predicted to be a superconductor; the values of superconducting transition temperature (*T*_c_) estimated by the McMillan–Allen–Dynes formula using *ab initio* calculation were 10, 2.9, and 13.3 K for Li,^10^ Na,^11^ and Ca^12^ intercalation cases, respectively. Superconductivity has been explained by the increase in the electron density of states (DOS) at the Fermi level, phonon softening owing to the screening effect,^10^ and the enhancement in electron phonon coupling.^10,12^

Furthermore, the change in electron and phonon energy band structures induced by strain provide tunability to the properties of MoS_2_.^13–15^ In the case of ultrathin MoS_2_, it is noteworthy that a strain of 6–11% can be induced with an average breaking strength of 15 ± 3 Nm^−1^.^16,17^ Enhanced superconductivity in a Na-intercalated bilayer with tensile strain was predicted by *ab initio* calculations.^11^ A relatively low *T*_c_ of 2.9 K at zero strain can be increased up to *T*_c_ = 10 K at 7% tensile strain, while superconductivity is suppressed under compressive strains.

In this study, we investigate the effect of strain on the superconductivity of Li-intercalated bilayer MoS_2_ for both compressive and tensile strain cases. In contrast to the Na-intercalation case, we discovered that superconductivity can be enhanced under both compressive and tensile strains. The emergence of superconductivity under compressive strain can be attributed to the significant softening of phonons at a specific ***q***-wave vector in the Brillouin Zone, which does not appear in the Na-intercalation case. Consequently, *T*_c_ is predicted to increase to 13.50 K at a 5.5% compressive strain.

The electron and phonon structures were calculated based on density functional theory (DFT)^18^ and density functional perturbation theory using Quantum Espresso^19^ combined with Wannier interpolation implemented in the Electron–Phonon Wannier (EPW)^20^ package. Local density approximation was used as an exchange-correlation functional with the norm-conserving pseudopotential^21,22^ distributed on the Quantum Espresso website. Furthermore, van der Waals interactions were considered using the Grimme-D2 method.^23^ A plane-wave basis set with an energy cutoff of 60 Ry was adopted to describe the electronic wave functions. The Methfessel–Paxton method^24^ with a smearing parameter of 0.01 Ry was used to obtain the charge density. A 24 × 24 × 1 Monkhorst–Pack^25^*k*-mesh was used for the electronic states and a 12 × 12 × 1 *q*-mesh for dynamical matrix calculations.

Superconductivity was studied based on the electron phonon coupling mechanism. First, we calculated the phonon linewidth as follows:1
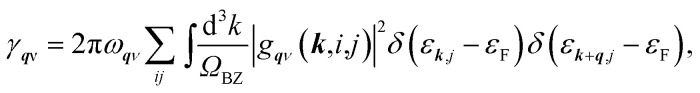
where *ω*_***q****ν*_ is the frequency of the phonon mode *ν* at wave vector ***q***, *Ω*_BZ_ is the volume of the Brillouin zone, and *g*_***q****ν*_(***k***,*i*,*j*) is the electron phonon (EP) matrix element. The electronic energy at branch *i* at wave vector ***k*** is given by *ε*_***k***,*i*_, and *ε*_F_ is the Fermi level. In practice, the EP matrix element is directly obtained using Quantum Espresso calculations. To compensate for the effect owing to the sparse sampling of the Brillouin zone, we used a finer 96 × 96 × 1 *k*-mesh to interpolate the Fermi surface.^26^ The two delta functions in eqn (1) were approximated by Gaussian functions with broadening width *σ*. After calculating various *σ* values and *k*- and *q*-mesh points, it was discovered that the combination of *σ* = 0.136 eV (=0.01 Ry) with a 96 × 96 × 1 *k*-mesh and a 12 × 12 × 1 *q*-mesh yielded converged values of the linewidths in the coarse grid level, and *σ* = 0.05 eV with dense 120 × 120 × 1 *k*- and *q*-meshes yielded converged values in the Wannier interpolated cases (see Section 4 in ESI†). The Eliashberg function and the electron phonon coupling (EPC) constant are defined in terms of the phonon linewidth as2
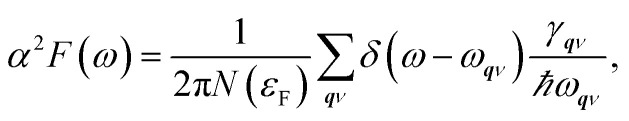
and3
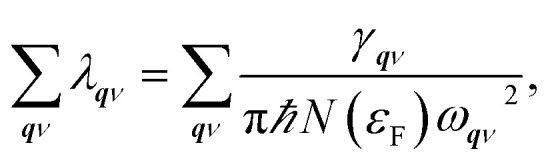
respectively, where *N*(*ε*_F_) is the density of states (DOS) at the Fermi level. The superconducting transition temperature *T*_c_ was estimated using the McMillan–Allen–Dynes formula,^27^4
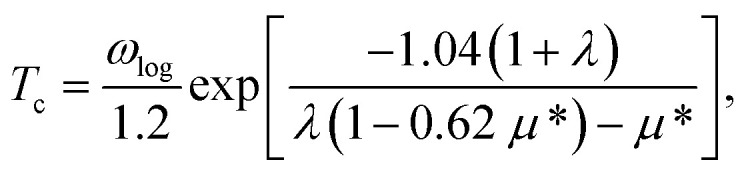
where5
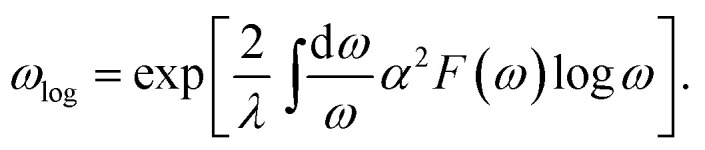


The estimate for *T*_c_ was further confirmed by evaluating the temperature dependence of the superconducting gap obtained by anisotropic Eliashberg equations.^28^ The convergence was achieved at 48 × 48 × 1 *k*-mesh and a 48 × 48 × 1 *q*-mesh (for details, see Section 5 in ESI†). Here, the Coulomb repulsion parameter μ* was set to 0.1. The nesting function, expressed as6
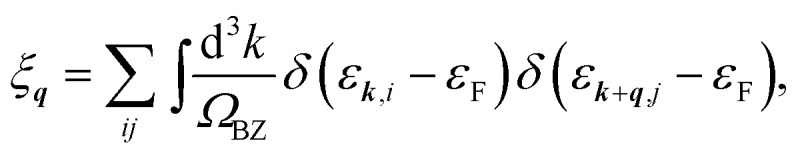


was analyzed to understand the relationship between the Fermi surface topology and the behavior of EP interaction.

Fig. 1 shows the model structure of the Li-intercalated bilayer MoS_2_ in this study. The stacking of MoS_2_ layers and the position of the intercalated Li have been reported as the most stable structure in a previous study.^10^ In the *z*-direction, we imposed a periodic boundary condition with a 15 Å vacuum layer. The in-plane lattice parameter of the Li-intercalated equilibrium bilayer structure was optimized to 3.16 Å, which agreed well with the experimental value of 3.16 Å in pristine bulk.^29^ A biaxial strain was induced by changing the in-plane lattice parameter up to ±8% with respect to the equilibrium one, whereas the out-of-plane direction was maintained. The atomic positions of all the strain-induced structures were relaxed with convergence thresholds of 0.5 meV Å^−1^ on the forces and 0.03 meV on the total energy. The relaxation caused the distances between the Mo-layer and S-layers to decrease under tensile strain and increase under compressive strain. This indicates a positive Poisson's ratio in the out-of-plane direction, which corresponds well to that reported in a previous study.^30^

**Fig. 1:**
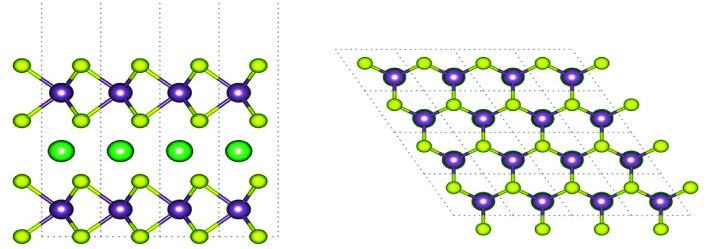
Side view (left) and top view (right) of the Li-intercalated bilayer MoS_2_ structure. Purple, yellow, and green spheres denote Mo, S, and Li, respectively.

The evolution of the electronic band structure under strain is presented in Fig. S1 ESI.† In all cases, Li intercalation caused a semiconductor–metal transition, and the original MoS_2_ conduction band crossed the Fermi level. The bands near the Fermi level comprised of Mo d_*xy*_, d_*x*_2_−*y*_2__, and d_*z*_2__ orbitals hybridized with S p-orbitals. Without strain, the two valleys, one located at the middle of the Γ–K path and the other at the middle of the Γ–M path, formed two Fermi surfaces with circular and lily-like shapes, respectively (Fig. 2b). As the compressive strain increased, the bandwidth increased owing to stronger hybridization, and the additional band crossed the Fermi level. This appeared as an excess small electron pocket, as shown in Fig. 2a. In contrast, as the tensile strain increased, the valleys that contributed to the Fermi surface differed. Instead of the valley in the middle of the Γ–M path, the valley at the *k* point formed an electron pocket. In addition, the Fermi surface formed by the valley in the middle of the Γ–K path is reduced. This resulted in a completely different Fermi surface compared to that in the zero-strain case (Fig. 2c). The above-mentioned changes in the Fermi surface topology are reflected in the nesting function shown in Fig. 2d–f. The value of the nesting function under compressive strain remained in the same order as that in the zero-strain case, while it increased under tensile strain.

**Fig. 2:**
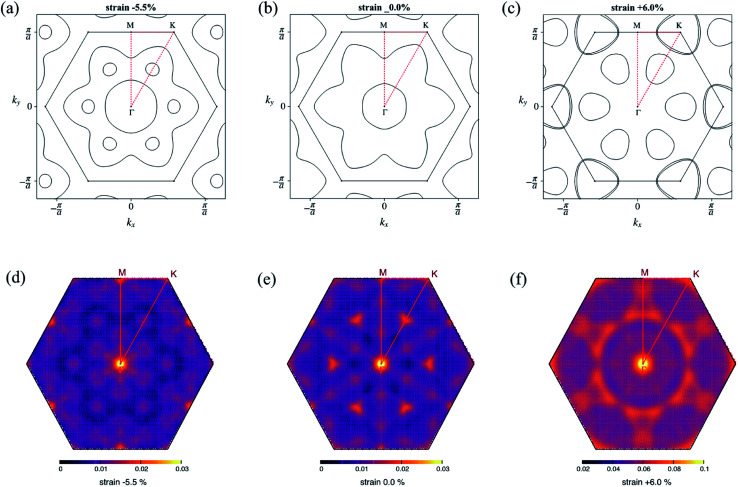
Fermi surface and nesting function at zero strain (b) and (e), −5.5% of compressive strain (a) and (d) and +6.0% of tensile strain (c) and (f) corresponding to the equilibrium state and where the maximum *T*_c_ appeared. The order of the nesting function at +6.0% strain was larger than that of the zero or compressive strain case. Note that the range of the color scale in (f) differs from those in (d) and (e).

Fig. 3 shows the strain dependence of the calculated superconductivity *T*_c_ together with those of the EPC constant (*λ*) and DOS at the Fermi level (*N*(*ε*_F_)). Thus, *T*_c_ can be increased by both tensile and compressive strains in the Li-intercalated bilayer MoS_2_ system. This point is consistent with the data estimated from the temperature dependence of the superconducting gap obtained by anisotropic Eliashberg equations^28^ shown in Fig. 4. The estimated *T*_c_ under zero strain was 0.46 K, which was considerably lower than that estimated in a previous study, that is, 10 K.^10^ This discrepancy may be caused by the different choice in lattice parameter or computation conditions (as shown in Fig. S4 in ESI†), as the *T*_c_ depends significantly on the choice of *k*- and *q*-meshes and Gaussian broadening *σ*.

**Fig. 3:**
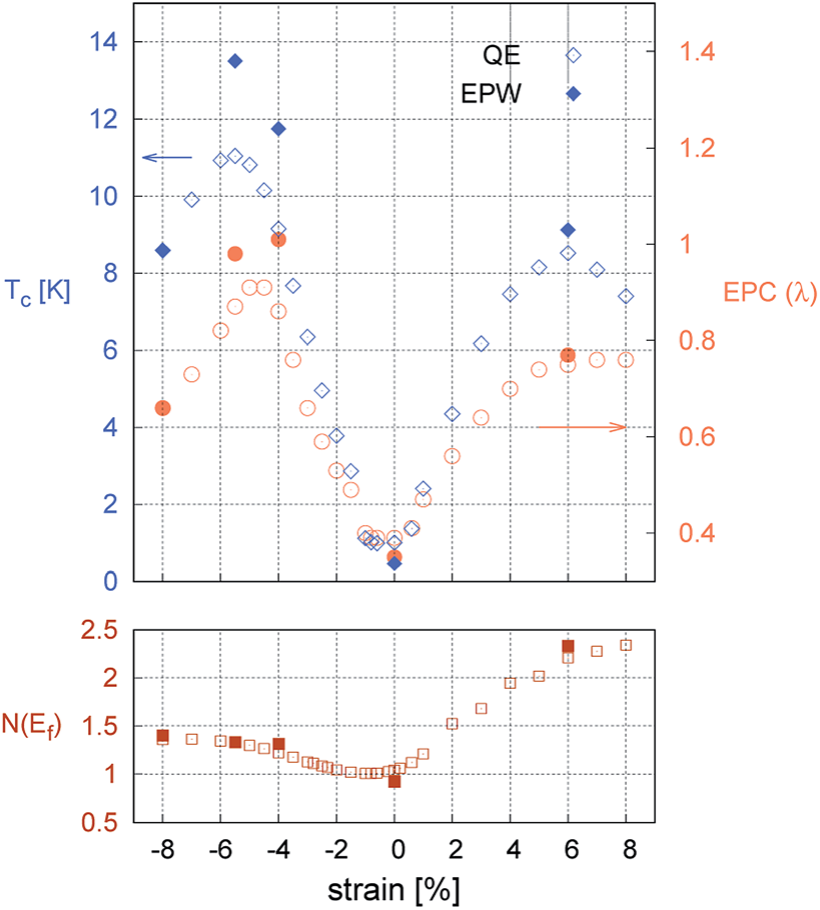
Change in transition temperature *T*_c_ [K], EPC constant (*λ*), and DOS at the Fermi level (*N*(*ε*_F_)) [states/spin/eV/unit cell] as a function of strain. Opened and closed dots refer to converged values in coarse *k*- and *q*-meshes from Quantum Espresso and in dense *k*- and *q*-meshes from EPW, respectively. Both results indicated well-converged conditions.

**Fig. 4:**
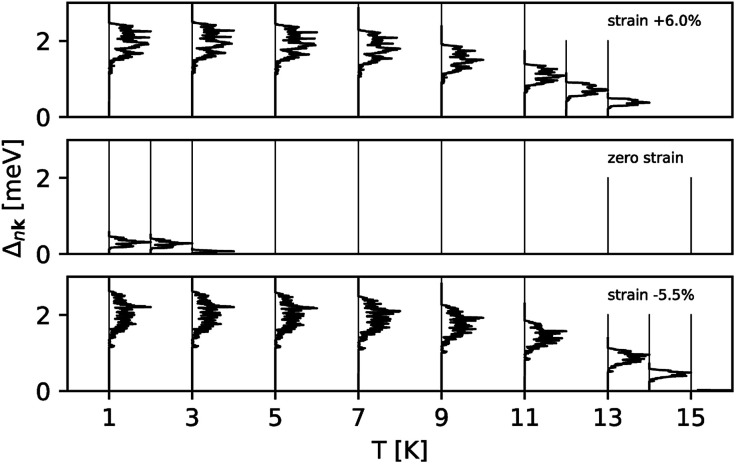
The superconducting gap obtained by the anisotropic Eliashberg equations as a function of temperature at strains 6, 0, and −5.5%.

At 6.0% tensile strain, the *T*_c_ reached a maximum at 9.12 K with an EPC constant *λ* = 0.77. The enhancement in *λ* can be partly explained by the strong intensity in the double-delta function term in eqn (1), which is of the same form as that of the nesting function. As shown in Fig. 2f, the change in the Fermi surface topology caused the nesting function to increase in the wide range of the Brillouin zone.

However, the explanation above based on the nesting function is not applicable to the compressive strain case where the intensity of the nesting function remains in the same order as that of the zero-strain case. Instead, we discovered a significant softening of the phonon, 
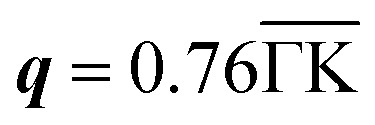
 accompanied by a large intensity in momentum and mode-resolved *λ*_***q****ν*_ (Fig. 5). Note that similar phonon behaviors have been observed in Li-intercalated bilayer SnS_2_ (ref. 31) and NaSn_5_,^32^ while significant enhancement in nesting function seen in these studies was not observed in the present study. This mode and the lowest phonon mode at the *k* point produce intensive peaks in the Eliashberg function and contribute 57% of the total EPC constant (*λ* = 0.98).

**Fig. 5:**
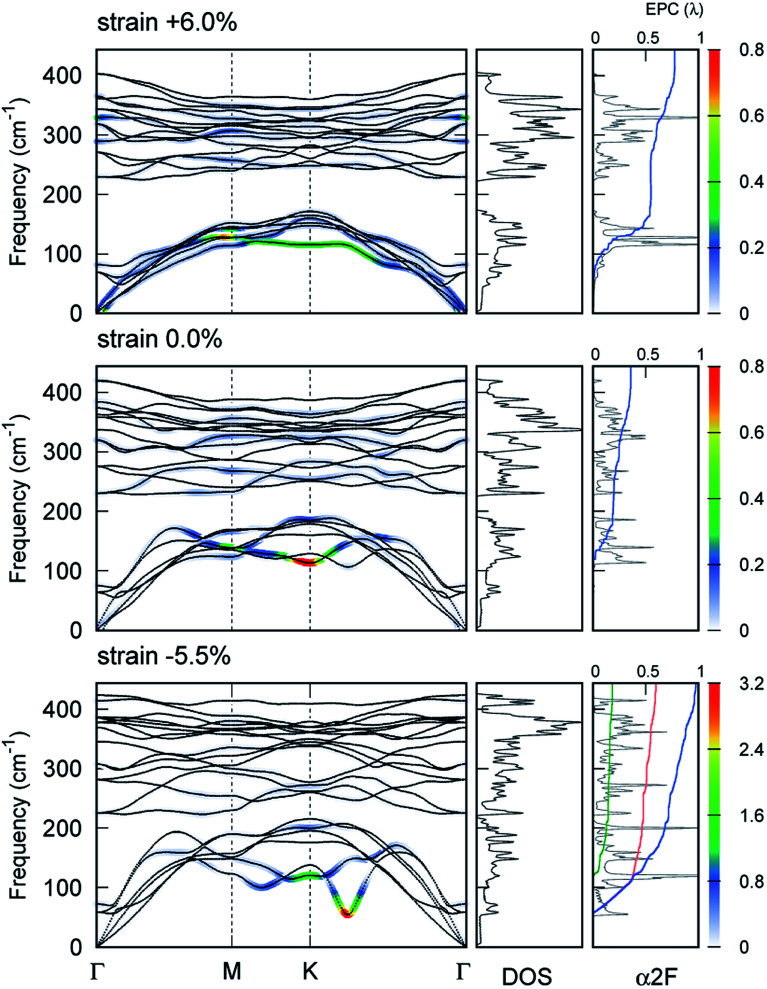
Phonon dispersion at zero strain (middle) and at the highest *T*_c_ with tensile (top) and compressive (bottom) strains together with phonon density of states (DOS) and Eliashberg spectral function (*α*^2^*F*). The color corresponds to the strength of the EPC constant. Integrated EPC as a function of frequency is shown (blue) together with an interband component (orange) and a pocket-involved intraband component (green) resolved through EPC.

We discovered that the appearance of the soft mode originated from the strong intrinsic EP matrix element. Considering the electron–phonon self-energy, Π(***q****ν*), the renormalized phonon frequency *ω*_***q****ν*_ can be described as^33^

7 (*ℏω*_***q****ν*_)^2^ = (*ℏω*^(0)^_***q****ν*_)^2^ + 2(*ℏω*^(0)^_***q****ν*_)|*g*_***q****ν*_|^2^Re[Π(***q****ν*)],

where *ω*^(0)^_***q****ν*_ is the phonon frequency of a bare ionic system without EP interactions, and Re[Π(***q****ν*)] represents the real part of the phonon's self-energy. We estimated eqn (7) by replacing |*g*_***q****ν*_|^2^ with the average of the square of the EP matrix elements over the Fermi surface,^34^ that is, 
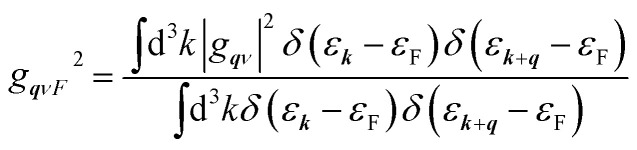
 and substituting 
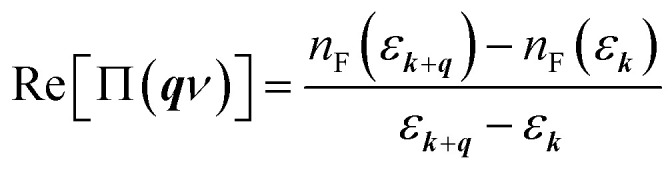
, where *n*_F_(*ε*_***k***_) is the Fermi–Dirac distribution function at the corresponding energy level, into the real part of the phonon's self-energy. The distribution of the factor |*g*_***q****ν*_|^2^Re[Π(***q****ν*)] shows an enhancement in the intrinsic EP matrix element at a specific momentum 
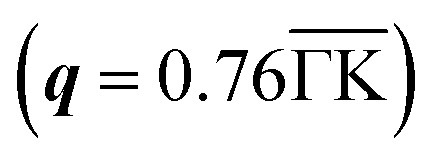
 and mode (see Section 6 in ESI†).

The soft mode above involves only a specific scattering process on the Fermi surface. In the compressive strain case, two bands crossed the Fermi level. We herein denote them as band (1) and (2). The decomposition of *λ*_***q****ν*_ into an intraband and interband shows that the interband process involving a small electron pocket that appeared at 
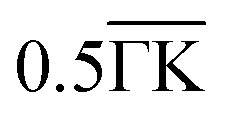
 formed by band (2) was important. The calculation results of each contribution are shown in Fig. 6b and c together with the nesting function limited to the d_*xy*_, d_*x*_2_−*y*_2__, and d_*z*_2__ orbitals of the Mo atoms (Fig. 6d and e) that primarily contributed to the EPC at the Fermi surface (see Section 8 in ESI†). *λ*_(*i*=*j*)_ has a similar distribution to the corresponding nesting function for the significantly high contribution from the wave vectors Γ, 
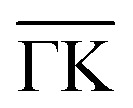
, and 
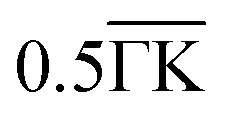
. The integrated values of *λ*_(*i*=*j*)_ for bands (1) and (2) were estimated to be 20% and 19% of the total EPC, respectively. Meanwhile, *λ*_(*i*≠*j*)_ was enhanced at 
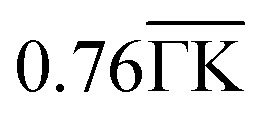
, where phonon softening occurred. The EPC integrated over the interband process becomes 61% of the total EPC. Therefore, interband scattering mediated by the soft mode dominates *T*_c_.

**Fig. 6:**
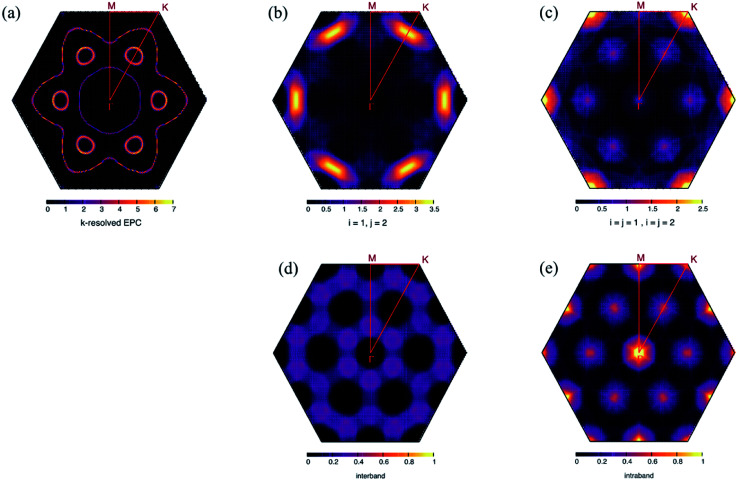
*k*-Resolved EPC constant (*λ*_*k*_) on the Fermi surface at −5.5% strain (a), *λ*_***q****ν*_ from interband scattering (b), *λ*_***q****ν*_ from intraband scattering (c), nesting function constructed from d_*xy*_, d_*x*_2_−*y*_2__ and d_*z*_2__ orbital of Mo atoms extracting the contribution from different band indexes (interband) (d), and the same band index (intraband) (e). The nesting functions were normalized to the same scale with 1.0 as the maximum of comparison.

An increase in EPC with phonon softening in TMDs is typically discovered in systems involving charge density waves (CDWs).^33,35–37^ Although CDWs have not been reported in doped MoS_2_ systems, their existence has been confirmed in other metal TMDs, such as NbSe_2_,^38^ TaSe_2_,^35^ and TiSe_2_.^39^ Recently, NbS_2_, a TMD, has been discovered to possess latent CDWs owing to its phonon behaviors and DOS at the Fermi level.^40^ Although a phase transition from an ordered structure to a distorted structure accompanied by a negative frequency did not appear in phonons, a significant phonon softening with enhanced intrinsic EP matrix elements under its metallic nature can be regarded as a precursor to the CDW transition. This seems consistent with a recent study on TaS_2_.^41^

In conclusion, we demonstrated that the superconductivity in Li-intercalated bilayer MoS_2_ can be enhanced by both compressive and tensile strains. In contrast to Na-intercalation, where the superconductivity is suppressed only under compressive strain, both tensile and compressive strains enhanced the superconductivity, whose *T*_c_ increased from 0.46 to 9.12 and 13.50 K under tensile and compressive strains, respectively. Under tensile strain, the enhancement was partly driven by an increase in the nesting function, which was related to the Fermi surface topology. However, in the compressive strain case, a significant phonon softening that occurred at a specific wave vector was found to be related to high intrinsic EP matrix elements. This indicates that the precursor state of CDW instability in layered materials can enhance superconductivity.

## Conflicts of interest

There are no conflicts of interest to declare.

## Supplementary Material

NA-002-D0NA00420K-s001
