# Supplementary material for: Straintronic effect for superconductivity enhancement in Li-intercalated bilayer MoS2
Source: Nanoscale Adv. 2020 Jul 6;2(8):3150–5. doi: 10.1039/d0na00420k (PMC9416899; doi:10.1039/d0na00420k)
Supplement: NA-002-D0NA00420K-s001 [file NA-002-D0NA00420K-s001.pdf]

Electronic Supplementary Material (ESI) for Nanoscale Advances.
This journal is © The Royal Society of Chemistry 2020

Supplementary Information
Straintronic effect for superconductivity enhancement in Li-intercalated bilayer MoS_2

Poobodin Mano [1], Emi Minamitani [2], and Satoshi Watanabe [1]

[1] Department of Materials Engineering, The University of Tokyo, 7-3-1 Hongo, Bunkyo, Tokyo 113-8656, Japan

[2] Institute for Molecular Science, South Laboratory Room413, 38 Nishigo-Naka, Myodaiji, Okazaki, Aichi 444-8585, Japan

1. Evolution of band structure under strain.

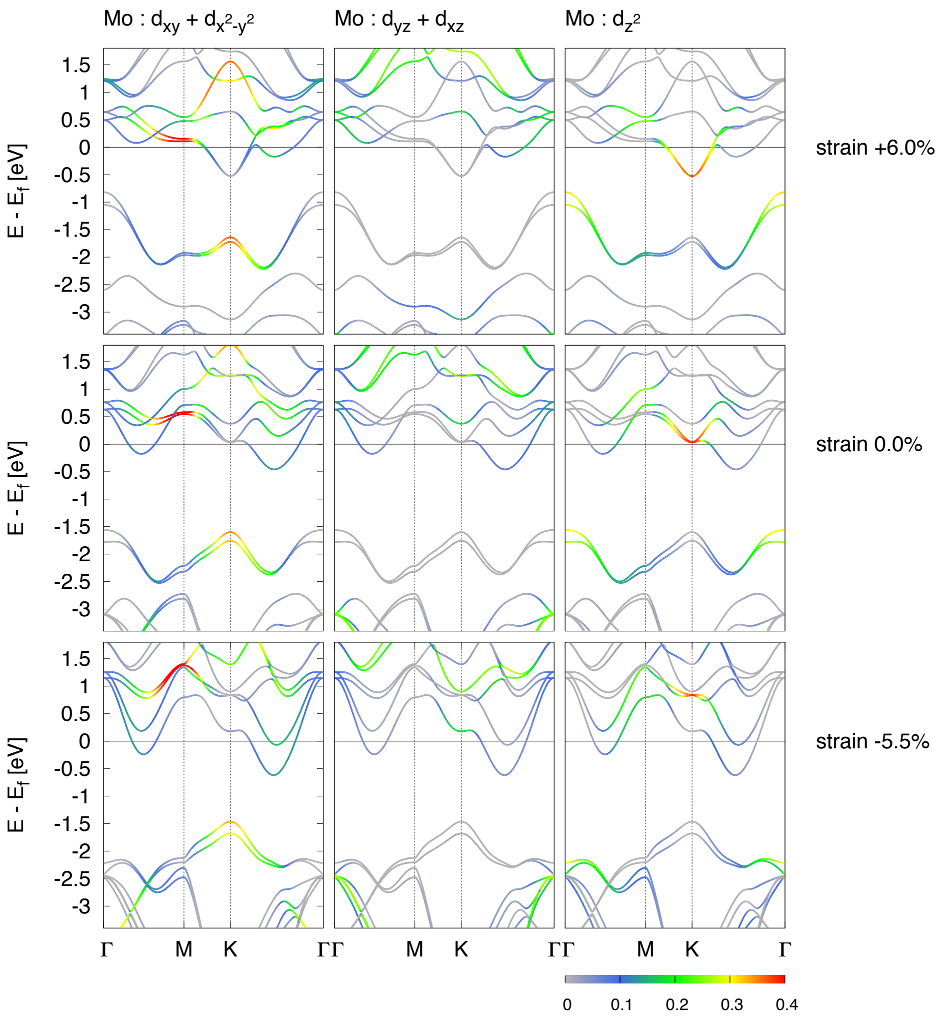

Fig. S1: Evolution of band structure at maximum T_c condition, i.e. strain +6.0% (top), 0.0% (middle), -5.5% (bottom). Band structures along high symmetry path ($\Gamma - M - K - \Gamma$) were colored with contribution from Mo d orbitals separated into degenerated pair $d_{xy} + d_{x^2-y^2}$ (left), $d_{yz} + d_{zx}$ (middle) and d_{z^2} (right). The Fermi level was set to zero.

2. Evolution of Fermi surface and nesting function under strains.

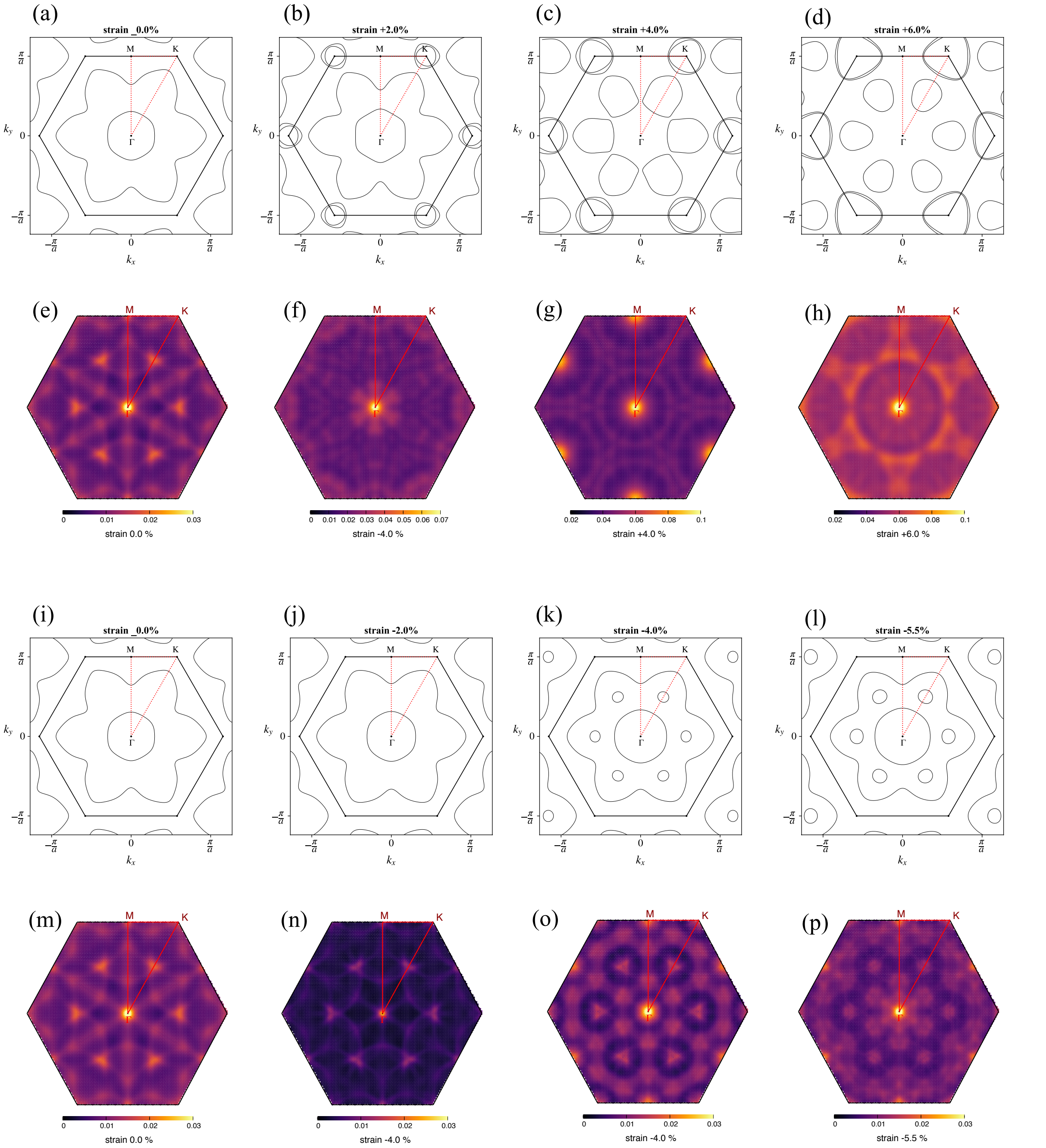

Fig. S2: The Fermi surface evolution under tensile strains (0% (a), +2.0% (b), +4.0% (c), +6.0% (d)) and compressive strains (0% (i), -2.0% (j), -4.0% (k), -5.5% (l)) together with corresponded nesting function of each Fermi surface.

3. Evolution of soft mode under compressive strain

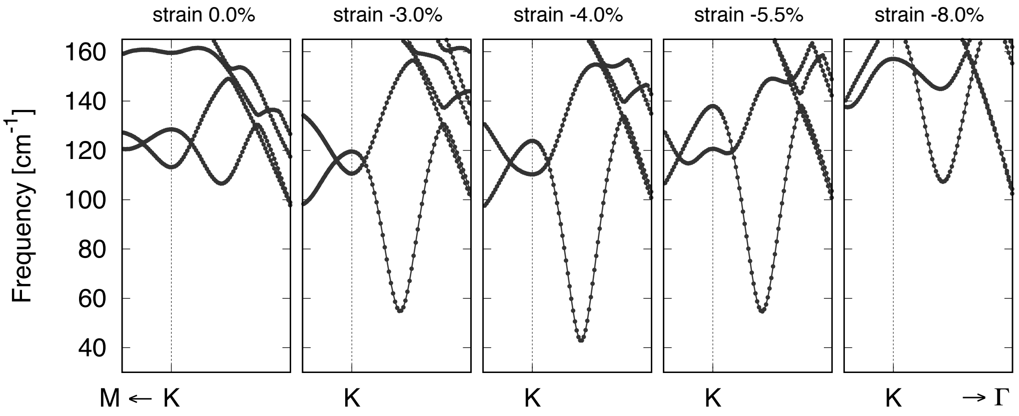

Fig. S3: Change of phonon frequency at $q = 0.76\overline{\Gamma K}$ as compressive strain increasing. Phonon frequency at $\boldsymbol{q} = 0.76\overline{\Gamma K}$ becomes lower as compressive strain increases. The softest frequency appeared at -4.0% strain.

4. Discussion on convergence of the double-delta functions.

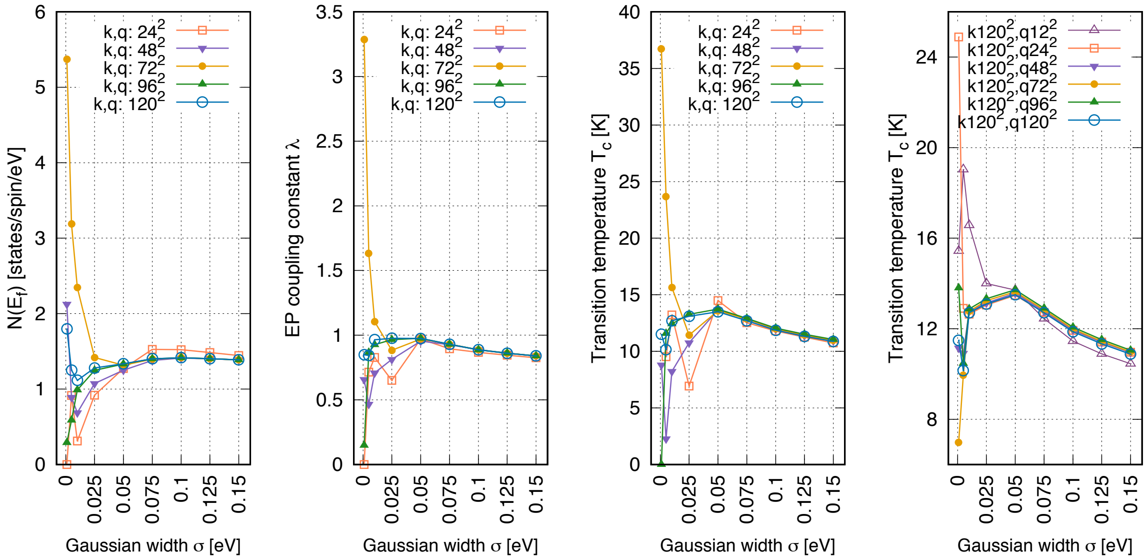

Fig. S4: The dependence of the density of states (DOS) at the Fermi level, EPC constant (λ) and transition temperature (T_c) on the value of σ and size of k-, q-points.

In practical estimation of the superconducting transition temperature based on the phonon linewidth defined in equation (8), the number of k-points, q-points and broadening width (σ), which is introduced in approximating delta functions in equation (8) by gaussians, are important parameters to determine the accuracy.

$$\gamma_{qv} = 2\pi\omega_{qv} \sum_{ij} \int \frac{d^3k}{\Omega_{BZ}} \left|g_{qv}(k,i,j)\right|^2 \delta\left(e_{q,i} - e_F\right)\delta\left(e_{k+q,j} - e_F\right) \quad \text{(S1)}$$

Thus, we performed the convergence test on these three parameters. For σ, a smaller value is better in principle because the gaussian functions are introduced to approximate delta functions. In practice, however, the balance between number of k-points and the value of σ should be considered in determining the value of σ. Figure S4 shows the dependence of the density of states (DOS) at the Fermi level, EPC constant (λ) and transition temperature (T_c) on the value of σ and size of k, q-points. We did the same manner in Quantum espresso (reasonable size of k-mesh) and EPW (dense k-mesh from Wannier interpolation) and here we show only the result from dense k-mesh under -5.5 % strain condition. As we can see in this figure, each value depends on number of k-points and σ significantly especially when σ is small. Similarly, when number of k-points is small, especially k = 24 × 24 × 1, the convergence respect to σ is slow and it does not converge into same value as the other size of k-mesh even at large σ. Then we concluded that k = 24 × 24 × 1 is not appropriate.

We found that 96 × 96 × 1k-mesh and 12 × 12 × 1 q-mesh with σ = 0.136 eV (= 0.01 Ry) is sufficient in Quantum espresso balanced with reasonable computational afford. In EPW level, a greater number of k, q points from Wannier interpolation, we use dense 120 × 120 × 1 for both k and q with σ = 0.05 eV to guarantee the convergence.

5. Temperature dependence of superconducting gap from anisotropic Eliashberg function

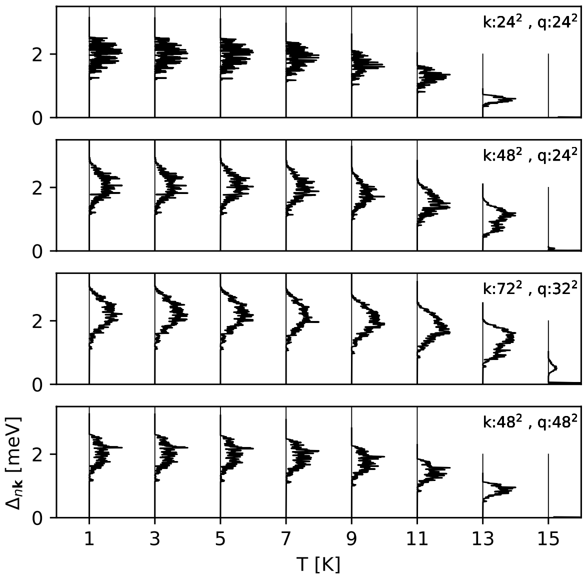

Fig. S5: Temperature dependence of superconducting gap on various number of k- and q-point (strain -5.5 % case).

In order to verify superconducting T_c from McMillan Allen-Dynes formula, we also calculated temperature dependence of superconducting gap from anisotropic Eliashberg function using EPW. The convergence test was done on several combination of number of k- and q-point. We found that k- and q-point dependence is small, and it converged rapidly. In this study we used $k = 48^2$ and $q = 48^2$ for superconducting calculation and the T_c was estimated to 15 K for -5.5% compressive strain case.

6. The factor $|g_{q\nu}|^2\mathrm{Re}[\Pi(q\nu)]$ and phonon renormalization

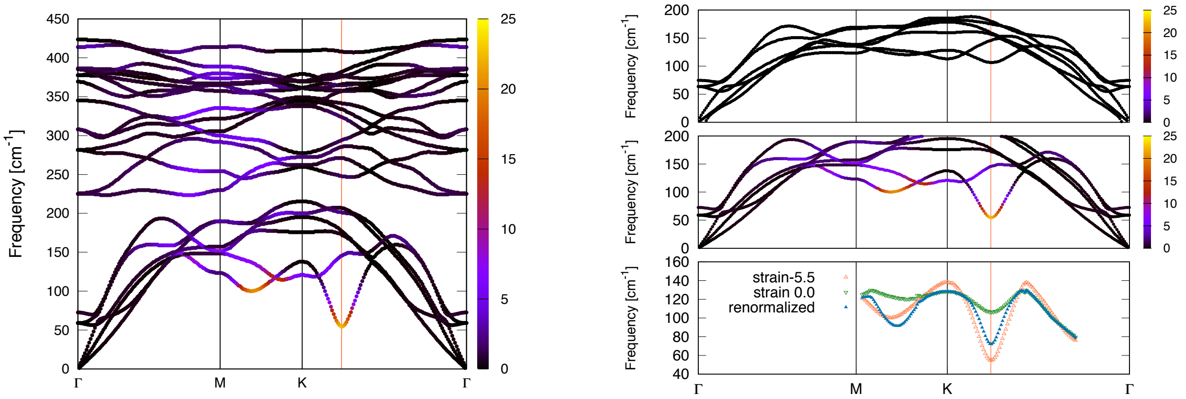

Fig. S6: Phonon dispersion at -5.5 % strain colored with the factor $|g_{q\nu}|^2\mathrm{Re}[\Pi(q\nu)]$ (left). Phonon renormalization (right) calculated from equation (9). Top subfigure is zero strain dispersion, middle subfigure is -5.5 % strain dispersion and bottom subfigure is result from equation (9)

Phonon frequency, in term of a result from the singularity of Dyson equation, is expressed by

$$(\hbar\omega_{q\nu})^2 = \left(\hbar\omega_{q\nu}^{(0)}\right)^2 + 2\left(\hbar\omega_{q\nu}^{(0)}\right)|g_{q\nu}|^2\mathrm{Re}[\Pi(q\nu)] \tag{S2}$$

where $\omega_{q\nu}^{(0)}$ is the phonon frequency of bare ionic system without EP interaction, $\omega_{\boldsymbol{q}\nu}$ is the frequency renormalized by including EP interaction and $\mathrm{Re}[\Pi(\boldsymbol{q}\nu)]$ is real part of phonon self-energy. The factor $|g_{q\nu}|^2\mathrm{Re}[\Pi(q\nu)]$ plays important role in determining phonon frequency. Here we visualized the factor into corresponded wave vector $\boldsymbol{q}$ and branch ν in phonon dispersion. We can see from the left figure, the softening due to the large EP matrix element occur at 3 different points in two lowest phonon modes. And the most conspicuous one is the softest mode at $q = 0.76\overline{\Gamma\mathrm{K}}$ which contributes largest to superconductivity.

We also calculated phonon renormalization from equation (9) by replacing frequency of bare ionic system ($\omega_{\boldsymbol{q}\nu}^{(0)}$) with zero strain dispersion. We already confirmed that EP interaction is significantly small at particular modes in zero strain dispersion (Figure S6: right top). There is discrepancy between renormalized curve and DFPT result for we did not consider strain effect to phonon frequency in phonon renormalization calculation here. The result, however, was in good tendency with DFPT curve.

7. EPC constant on phonon dispersion with each scattering process.

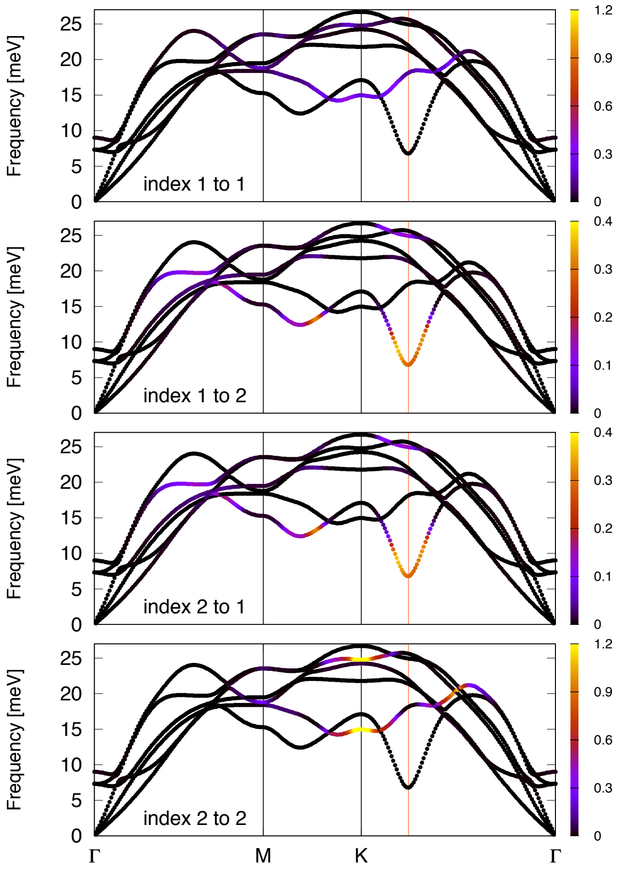

Fig. S7: Electron phonon coupling in each scattering process, i.e. interband and intraband scattering.

Electron phonon coupling (EPC) constant is a result from integration over electron phonon (EP) matrix element which can be expressed by equation below.

$$g_{q\nu}(k,i,j) = \left(\frac{\hbar}{2M\omega_{q\nu}}\right)^{1/2} \left\langle \psi_{i,k} \left| \frac{dV_{SCF}}{du_{q\nu}} \cdot \varepsilon_{q\nu} \right| \psi_{j,k+q} \right\rangle. \tag{S3}$$

EP matrix element is labeled with 5 parameters, i.e. phonon wave vector q and branch ν, electronic wave vector $\boldsymbol{q}$ at band index i for initial state and j for final state. Decomposed EPC constant on each transition process can be obtained by constraining band index i, j in summation over all these parameters.

Figure S7 shows electron phonon coupling constants on corresponding $\boldsymbol{q}$ wave vector colored by the strength of EPC constant $\lambda_{q\nu}$. The same result for all Brillouin Zone was shown in the paper. Band index 2 contributes largely to EPC. EP interaction involved by soft mode $\boldsymbol{q} = 0.76\overline{\Gamma \mathrm{K}}$ occurs through only interband scattering process. Thus, the appearance of band index 2 on the Fermi surface is important for EPC enhancement under compressive strain induction.

8. Orbital resolved Fermi surface

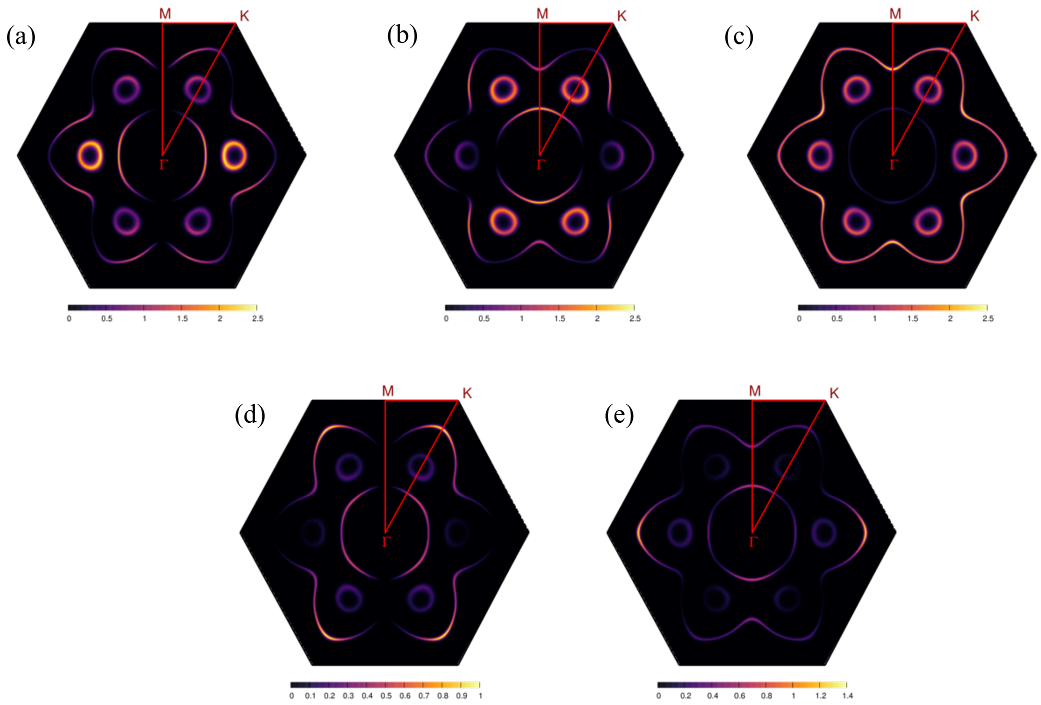

Fig. S8: Contribution from $d_{xy}, d_{x^2-y^2}, d_{z^2}$ is larger than that of d_{yz} and d_{xz} orbital at entire Fermi surface, especially at the pockets (middle of $\Gamma - \mathrm{K}$). For $d_{xy}, d_{x^2-y^2}$ orbital, the strong value corresponded to strong EPC (λ_k) distribution on Fermi surface, indicate the importance of these orbital to EP interaction in Li-intercalated bilayer MoS_2.
